# Multi-modal neo-adjuvant anti-obesity medications may be more effective than medically supervised weight loss or GLP-1 therapy alone in preparing BMI≥70 patients for metabolic surgery

**DOI:** 10.1038/s41366-025-01798-2

**Published:** 2025-06-02

**Authors:** Michael Kachmar, Florina Corpodean, Iryna Popiv, Kyle B. LaPenna, Denise M. Danos, Michael W. Cook, Brian A. Saunders, Jean J. Domercant, Vance L. Albaugh, Philip R. Schauer

**Affiliations:** 1 Pennington Biomedical Research Center at Louisiana State University, Baton Rouge, LA USA; 2 Department of Surgery, Louisiana State University Health Sciences Center, New Orleans, LA USA; 3 Our Lady of the Lake, Franciscan Missionaries of Our Lady Health System, Baton Rouge, LA USA; 4 Department of Behavioral & Community Health, Louisiana State University Health Sciences Center, New Orleans, LA USA; 5 University Medical Center, New Orleans, LA USA; 6 Metamor™ Metabolic Institute, Pennington Biomedical Research Center, Baton Rouge, LA USA

**Keywords:** Weight management, Obesity

## Abstract

**Background/objectives:**

Optimizing patients with a body mass index (BMI) ≥ 70 kg/m² for metabolic surgery (MS) is challenging. However, pre-operative weight loss may be important for improving the safety of MS for these high-risk patients. Multi-modal anti-obesity medications (mmAOM) may enhance preoperative weight loss compared to non-pharmacologic medically supervised weight loss (NP-MSWL) or glucagon-like peptide-1 receptor agonist monotherapy (Mono-GLP-1) alone.

**Subjects/methods:**

This retrospective study analyzed 113 patients with BMI ≥ 70 kg/m² at a single metabolic disease treatment institute.

**Interventions/methods:**

Patients were categorized into NP-MSWL (*n* = 13), Mono-GLP-1 (*n* = 54), and mmAOM (*n* = 46) groups. The primary outcome was mean percent total body weight loss (%TBWL). Secondary outcomes included %TBWL across time intervals (0–23, 23–51, 51–88, and 88+ weeks).

**Results:**

The mmAOM group achieved the highest average - 13.07% - and median (9.93% [5.57–14.29]) %TBWL; followed by Mono-GLP-1 (5.58% [0.98–10.19]); and NP-MSWL (5% [2.97–7.02]). Significant differences among groups were confirmed by Kruskal-Wallis test (*p* = 0.0047). The highest median %TBWL was at 51–88 weeks (10.25 [6.49–16.45]) (*p* = 0.0093).

**Conclusions:**

mmAOM treatment yields the highest %TBWL, especially within the first 51 weeks of preoperative preparation, demonstrating superior efficacy over Mono-GLP-1 and NP-MSWL in patients with BMI ≥ 70 kg/m². These findings suggest that incorporating mmAOM in preoperative protocols could optimize weight loss and improve surgical outcomes for high BMI patients.

## Introduction

While metabolic surgery (MS) has long been proven to be the most effective and durable treatment option for individuals with obesity and obesity related disease [1, 2], the explosion of increasingly effective anti-obesity medication (AOM) classes, namely glucagon-like peptide-1 receptor agonists (GLP-1-RAs) and other enteroendocrine based therapy, have contributed to a flurry of renewed public interest in both the medical and surgical treatment of obesity.

Traditionally, preoperative MS protocols include weight loss strategies, especially in patients with a very high body mass index (BMI) to improve perioperative safety and technical feasibility for minimally invasive surgery. Prior to the introduction of GLP-1RAs, however, few pharmaceutical options demonstrated weight loss efficacy consistently achieving > 5% weight loss which is the threshold for measurable clinical benefit [3]. Consequently, the development of effective neo-adjuvant AOM protocols was limited. The question that remains is, how can these medications be optimally used in the neo-adjuvant MS setting in preparation for surgery?

It is well-established that surgery carries increased risks in populations with very high BMIs [4–8], especially those with a body mass index (BMI) ≥ 70 kg/m^2^. However, it is also known that pre-operative weight can be decreased with AOMs as well as other less durable non-pharmacologic means (e.g. behavioral changes, dietary modifications, and increased physical activity). In the current era of GLP-1-RAs, there is uncertainty about the most effective medication, or combination of medications, for pre-operative weight loss. By retrospectively analyzing patients with a BMI ≥ 70 kg/m^2^ at a single institution, this study sought to determine the most effective number, class, and length of neo-adjuvant therapies for achieving robust pre-operative weight loss for this specific population. It is anticipated that findings would provide valuable insights into optimizing pre-operative care and enhancing surgical outcomes for high BMI patients.

## Methods

Patients with a BMI ≥ 70 kg/m^2^ seeking care between September 2019 and the latest date August 2023 at Metamor^TM^ Metabolic Institute in Baton Rouge, Louisiana, were included in the study cohort (*n* = 226). Exclusions were: (1) patients who did not complete at least one in-system follow-up visit, (2) did not participate in the medically supervised weight loss program, or (3) were otherwise lost to follow-up. After exclusions, a total of 113 cases were included in full analysis.

Patients were categorized into three groups based on their treatment modality: (1) Non-pharmacologic medically supervised weight loss (NP-MSWL), which included patients who made diet and exercise changes without any medication; (2) Glucagon-like peptide-1 receptor agonist monotherapy (Mono-GLP-1), which included patients who were treated with a single GLP-1-RA (e.g. liraglutide, semaglutide, dulaglutide, tirzepatide) and no other medications; and (3) mmAOM, which included patients who were treated with a combination of medications, either a GLP-1-RA with one or more of phentermine, topiramate, orlistat, or metformin, or a combination of these medications without a concurrent GLP-1-RA. Both the Mono-GLP-1 and mmAOM groups received the same medically supervised weight loss recommendations and monitoring as the NP-MSWL group, in addition to their pharmacotherapy.

All sensitive health information was collected and stored on institutional servers in a HIPPA compliant manner utilizing a secure REDCap project (Vanderbilt University, Nashville, TN, US) [9]. De-identified data was cleaned and analyzed utilizing R (v4.3.1 – R Foundation for Statistical Computing; Vienna, Austria) [10].

The primary outcome was percent total body weight loss (%TBWL) from the initial presenting weight to the weight at the date of surgery or at the maximum length of follow-up, whichever occurred first. A sub-analysis was conducted based on the quartile duration of supervised weight loss, divided into four groups: 0–23, 23–51, 51–88, and more than 88 weeks. Additionally, baseline co-morbidities and patient characteristics were recorded and assessed.

In addition to the primary analysis comparing weight loss outcomes across treatment groups and time, two post hoc analyses were conducted. The first stratified weight loss outcomes by diabetic vs. non-diabetic status to evaluate potential differences in treatment response. The second categorized patients based on whether they had ever received a second-generation GLP-1 receptor agonist (e.g., semaglutide, tirzepatide) at any point during treatment versus only receiving a first-generation GLP-1 receptor agonist (e.g., liraglutide, dulaglutide).

Percent Total Body Weight Loss (%TBWL) was calculated as the difference between initial weight and post-treatment/preoperative weight, divided by initial weight, multiplied by 100. Percent Excess Weight Loss (%EWL) was calculated as weight loss divided by the difference between initial weight and ideal weight, multiplied by 100, where ideal weight is based on a BMI of 25 kg/m^2^.

A Kruskal-Wallis was utilized for the analysis of %TBWL and other continuous variables, including age, BMI, and treatment length. Categorical variables, such as comorbidities and gender, were analyzed using either the Chi-squared test or Fisher’s Exact test, depending on the cell counts. Post-hoc pairwise comparisons of %TBWL were conducted using Wilcoxon rank-sum tests for the primary analysis. Statistical significance was set at a *p*-value of < 0.05. All results are reported as: median [interquartile range (IQR)], unless otherwise noted.

This study was conducted in accordance with ethical standards and guidelines, and the research protocol was reviewed and approved by the Institutional Review Board (IRB) at Pennington Biomedical Research Center. Informed consent was waived due to the retrospective design and minimal risk to participants.

## Results

The baseline characteristics of the study population are summarized in Table 1. The study included a total of 113 participants, with 13 in the NP-MSWL group, 54 in the Mono-GLP-1 group, and 46 in the mmAOM group. Median age was similar (*p* = 0.134) across the groups, with an overall median of 41.2 years [27.2–55.2], as was median pre-treatment BMI (*p* = 0.376) with a median of 80.4 (min 71- max 118). Sex distribution showed no significant differences, with 18.6% male and 76.1% female participants overall (*p* = 0.159 and *p* = 0.225, respectively).

**Table 1:** Baseline patient characteristics.

	Overall	NP-MSWL	Mono-GLP-1	mmAOM	*p*-value
Total, *n*	113	13	54	46	
Age, median [IQR]	41.2 [27.2–55.2]	39.1 [25.1–53.1]	42.9 [30.2–55.7]	39.6 [25.6–53.6]	0.134
BMI, median (Min - Max)	80.4 (71–118)	79.42 (72–97)	81.69 (71–118)	79.04 (71–94)	0.376
Treatment Length, [IQR]	51.14 [23.14–88.29]	36.21 [17.89–83.43]	50 [23.14–69.86]	57.43 [36.18–107.93]	0.118
Sex, *n* (%)					
Male	21 (18.6%)	2 (15.4%)	14 (25.9%)	5 (10.9%)	0.159
Female	86 (76.1%)	11 (84.6%)	37 (68.5%)	38 (82.6%)	0.225
Diabetes mellitus, n (%)	69 (61.1%)	4 (30.8%)	37 (68.5%)	28 (60.9%)	0.047
Hypertension, *n* (%)	88 (77.9%)	10 (76.9%)	45 (83.3%)	33 (71.7%)	0.382
Hyperlipidemia, *n* (%)	33 (29.2%)	3 (23.1%)	20 (37%)	10 (21.7%)	0.235
Sleep Apnea, *n* (%)	71 (62.8%)	7 (53.8%)	31 (57.4%)	33 (71.7%)	0.260
GERD, *n* (%)	29 (25.7%)	3 (23.1%)	15 (27.8%)	11 (23.9%)	0.953
COPD, *n* (%)	9 (8%)	1 (7.7%)	3 (5.6%)	5 (10.9%)	0.697
CAD, *n* (%)	2 (1.8%)	0 (0.0%)	1 (1.9%)	1 (2.2%)	1
Heart Failure, *n* (%)	13 (11.5%)	1 (7.7%)	9 (16.7%)	3 (6.5%)	0.256
Atrial Fibrillation, *n* (%)	8 (7.1%)	1 (7.7%)	4 (7.4%)	3 (6.5%)	0.999
Osteoarthritis, *n* (%)	23 (20.4%)	3 (23.1%)	12 (22.2%)	8 (17.4%)	0.797
Anxiety, *n* (%)	25 (22.1%)	3 (23.1%)	13 (24.1%)	9 (19.6%)	0.810
Depression, *n* (%)	39 (34.5%)	7 (53.8%)	17 (31.5%)	15 (32.6%)	0.295

*NP-MSWL* Non-Pharmacologic Medically Supervised Weight Loss, *Mono-GLP-1* Glucagon-Like Peptide-1 Receptor Agonist Monotherapy (Mono-GLP-1), *AOM* Anti-Obesity Medication, *BMI* Body Mass Index. Age recorded in years, *BMI* recorded in kg/m², Treatment length recorded in weeks.

With respect to obesity associated diseases among the groups, there were differences observed in the prevalence of diabetes mellitus that was more common in the Mono-GLP-1 group (68.5%) compared to the NP-MSWL group (30.8%) and the mmAOM group (60.9%) (*p* = 0.047). Interestingly, no significant differences were detected in prevalence of hypertension (*p* = 0.382), hyperlipidemia (*p* = 0.235), sleep apnea (*p* = 0.260), GERD (*p* = 0.953), COPD (*p* = 0.697), CAD (*p* = 1.000), heart failure (*p* = 0.256), atrial fibrillation (*p* = 0.999), osteoarthritis (*p* = 0.797), anxiety (*p* = 0.810), or depression (*p* = 0.295) among the treatment groups.

**Fig. 1:**
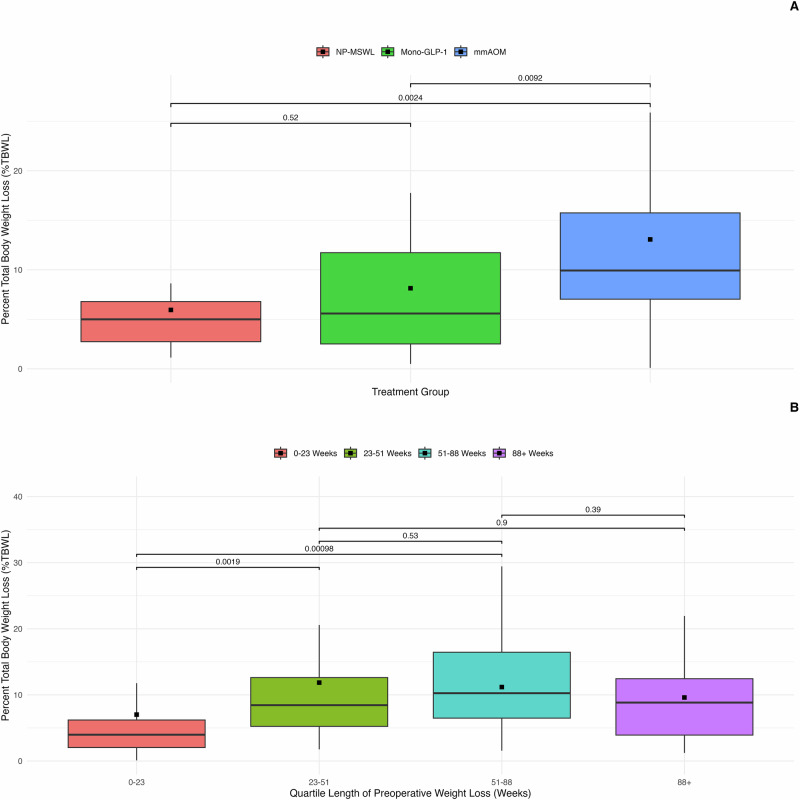
Percent Total Body Weight Loss (%TBWL) by Treatment Group and Treatment Length Intervals. **A** Box plots representing the distribution of %TBWL by treatment group: non-pharmacologic medically supervised weight loss (NP-MSWL) glucagon-like peptide-1 receptor agonist monotherapy (Mono-GLP-1) and multi-modal anti-obesity medication (mmAOM). Kruskal–Wallis test *p* = 0.0047 annotated *p* values represent post-hoc pairwise comparisons using Wilcoxon rank-sum tests. **B** %TBWL across four treatment length intervals: 0–23 weeks, 23–51 weeks, 51–88 weeks, and 88+ weeks. Kruskal–Wallis test *p* = 0.0022 annotated *p* values represent post-hoc pairwise comparisons using Wilcoxon rank-sum tests. In both panels boxes represent the interquartile range (IQR) horizontal black lines denote median %TBWL black squares indicate mean %TBWL and whiskers extend to the minimum and maximum values within the 95% confidence interval (CI).

Overall and treatment group reductions in body mass and weight are detailed in Table 2. Analysis of %TBWL by treatment modality demonstrated significant differences among the groups (Fig. 1A**)**. The mmAOM group achieved the highest %TBWL, followed by the Mono-GLP-1 and NP-MSWL groups (*p* = 0.0047). NP-MSWL was found to have the lowest %TBWL with a mean percentage weight loss of 5.95% (median, 5% [2.97–7.02]). Mono-GLP-1 therapy showed an increased mean %TBWL of 8.14% (median, 5.58% [0.98–10.19]). The mmAOM group achieved the highest mean %TBWL at 13.07% (median, 9.93% [5.57–14.29]). Additionally, analysis of %TBWL by quartile length of treatment indicated the highest %TBWL was appreciated at 51–88 weeks. (Fig. 1B) (*p* = 0.0022). Median %TBWL for each time interval was as follows: 0–23 weeks: 3.98% [2.04–6.2] (*n* = 29); 23–51 weeks: 8.44% [5.25–12.62] (*n* = 28); 51–88 weeks: 10.25% [6.49–16.45] (*n* = 28); and 88+ weeks: 8.84% [3.92–12.45] (*n* = 28).

**Table 2:** Weight loss outcomes by treatment group.

	Overall	NP-MSWL	Mono-GLP-1	mmAOM	*p*-value
Total, n	113	13	54	46	
%TBWL, median [IQR]	7.81 [3.57–12.06]	5 [2.97–7.02]	5.58 [0.98–10.19]	9.93 [5.57–14.29]	0.005
Δ BMI, median [IQR]	6.21 [2.09–10.33]	5 [3.94–6.06]	4.93 [0.72–9.13]	8.48 [5.01–11.96]	0.092
Δ kg, median [IQR]	15.5 [4.14–26.86]	7.8 [2.55–13.05]	12.15 [–0.03–24.33]	19 [8.91–29.09]	0.017
%EWL, median [IQR]	11.4 [5.06–17.75]	7.2 [4.54–9.87]	8.33 [1.98–14.67]	14.65 [8.82–20.49]	0.004

*NP-MSWL* Non-Pharmacologic Medically Supervised Weight Loss, *Mono-GLP-1* Glucagon-Like Peptide-1 Receptor Agonist Monotherapy (Mono-GLP-1), *mmAOM* Multi-Modal Anti-Obesity Medication, *BMI* Body Mass Index. All data are presented as mean [95% confidence interval]. Percent Total Body Weight Loss (%TBWL) calculated as: %TBWL = ((Presentation Weight − Post-treatment Weight) / Initial Weight) × 100. Percent Excess Weight Loss (%EWL) calculated as: %EWL = (Weight Loss) / (Presentation Weight − Ideal Weight) × 100. Ideal weight was based on a BMI of 25 kg/m².

**Fig. 2:**
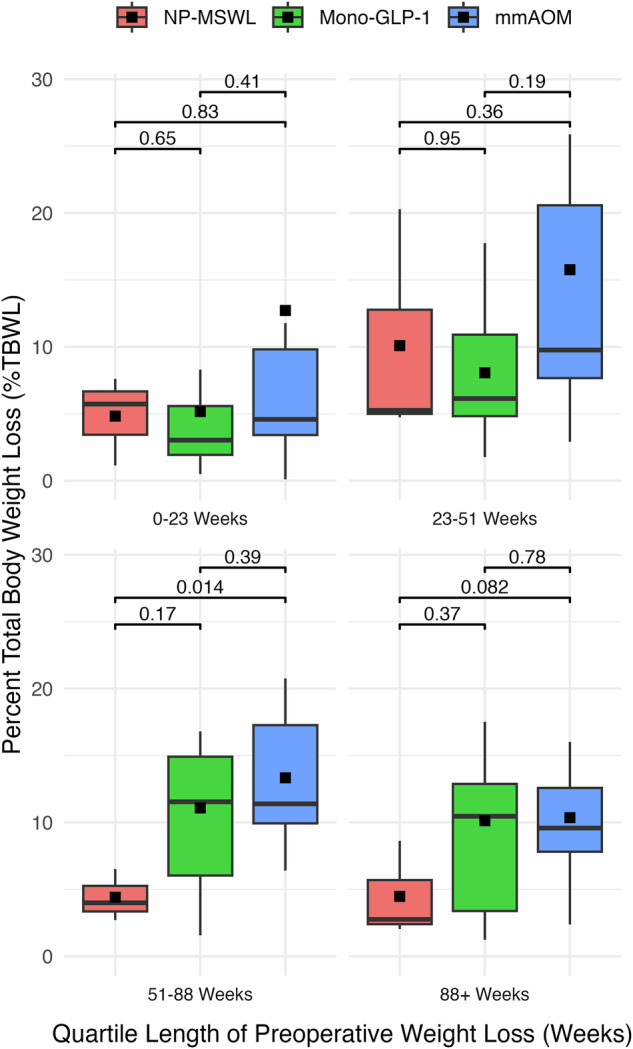
Interaction between treatment modality and duration on percent total body weight loss (%TBWL). Box plots illustrating percent total body weight loss (%TBWL) broken out by treatment modality and duration of treatment over four time intervals: 0–3 weeks, 23–51 weeks, 51–88 weeks, and 88+ weeks. The treatment groups include Non-Pharmacologic Medically Supervised Weight Loss (NP-MSWL), Glucagon-Like Peptide-1 Receptor Agonist Monotherapy (Mono-GLP-1), and Multi-Modal Anti-Obesity Medication (mmAOM). The boxes represent the interquartile range (IQR), the horizontal black lines denote the median %TBWL, and the black squares indicate the mean %TBWL. The whiskers extend to the minimum and maximum values within the 95% confidence interval (CI). Kruskal-Wallis test *p* = 0.0093; annotated *p*-values represent post-hoc pairwise comparisons nested by quartile length using Wilcoxon rank-sum tests.

When %TBWL was broken out by both modality and length of treatment it continued to reach significance (*p* = 0.0093), with both mmAOM and Mono-GLP-1 groups demonstrating their highest %TBWL ~ 51 weeks (Fig. 2). However, the only significant pairwise comparison nested by quartile length was between mmAOM and NP-MSWL – consistent with our overall and pair-wise tests between modalities (Fig. 1A**)**. The NP-MSWL group demonstrated a median %TBWL of 5.73% [3.43–6.67] at 0–23 weeks, 5.26% [5–12.77] at 23–51 weeks, 3.99% [3.35–5.26] at 51–88 weeks, and 2.75 [2.4–5.69] at 88+ weeks (Fig. 2). Mono-GLP-1 group showed a median %TBWL of 3.03% [1.93–5.57] at 0–23 weeks, 6.14% [4.82–10.91] at 23–51 weeks, 11.54% [6.03–14.91] at 51–88 weeks, and 10.46% [3.38–12.88] at 88+ weeks. The mmAOM group showed a median %TBWL of 4.58% [3.41–9.81] at 0–23 weeks, 9.77% [7.67–20.58] at 23–51 weeks, 11.39% [9.93–17.27] at 51–88 weeks, and 9.58% [7.81–12.58] at 88+ weeks.

**Fig. 3:**
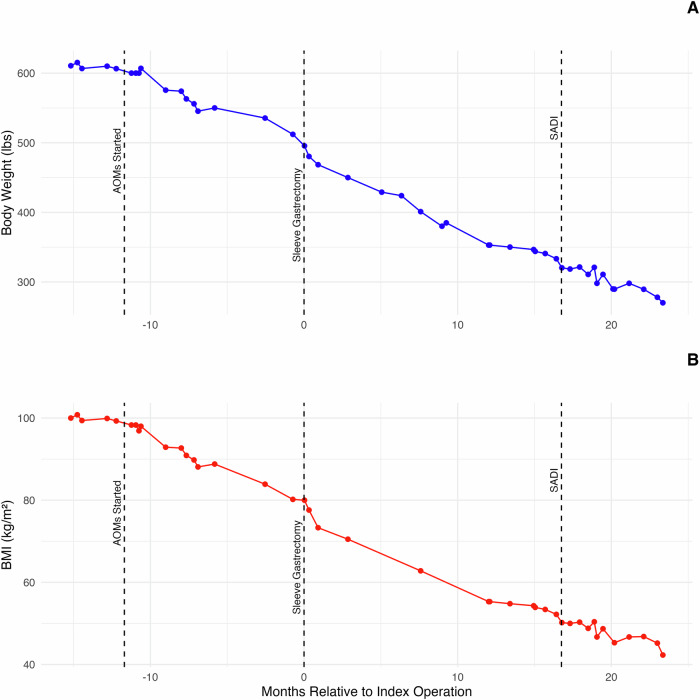
Representative patient weight and BMI trajectory with neo-adjuvant AOM and operative intervention. Figure illustrating the trajectory of a representative patient’s body weight (**A**) and BMI (**B**) over time in relation to key interventions. The timeline was centered around the first-stage operation (Sleeve Gastrectomy), marked as month 0. Body weight and BMI were optimized prior to surgery using neo-adjuvant anti-obesity medication (AOM) therapy, as indicated by the “AOMs Started” line. Post-operatively, continued weight and BMI reductions were demonstrated. The dashed vertical lines indicate significant dates for the start of AOM therapy, the first-stage operation, Sleeve Gastrectomy, and the second-stage operation, single anastomosis duodenal bypass (SADI).

The weight and BMI trajectory of a representative patient (initial BMI of 101 kg/m^2^) from this cohort (Fig. 3) illustrates the dynamic changes in body weight throughout the course of anti-obesity medication (AOM) therapy, sleeve gastrectomy (SG), and subsequent planned conversion to a single anastomosis duodeno-ileal bypass (SADI).

Among the study population, diabetes prevalence differed across treatment groups, with the highest proportion in the Mono-GLP-1 group (68.5%) compared to the mmAOM (60.9%) and NP-MSWL (30.8%) groups. Given the known impact of diabetes and metabolic disease on weight loss response, a post hoc analysis stratified outcomes by diabetic vs. non-diabetic status (Supplementary Table 1). While both groups achieved weight loss, non-diabetic patients demonstrated higher median %TBWL across many treatment modalities.

Given the differences in efficacy between first- and second-generation GLP-1 receptor agonists, a post hoc analysis categorized patients based on whether they had ever received a second-generation GLP-1-RA (e.g. semaglutide or tirzepatide during their treatment course). Patients who received a second-generation GLP-1-RA demonstrated a greater median %TBWL compared to those who only received first-generation agents in many treatment groupings (Supplementary Table 2).

## Discussion

Metabolic surgery remains highly effective for treating obesity, but new potent AOMs have augmented interest in both medical and surgical treatment approaches. This study aimed to better determine effects of different neo-adjuvant therapies for pre-operative weight loss in patients with BMI ≥ 70 kg/m^2^, a common factor aiming to improving surgical outcomes for high BMI patients. Specifically, we evaluated the efficacy of mmAOM compared to NP-MSWL and Mono-GLP-1 therapy in achieving preoperative weight loss in patients with a BMI ≥ 70 kg/m². Our findings suggest that mmAOM therapy significantly improves %TBWL in this high-risk population preparing for metabolic surgery. More specifically, mmAOM therapy appears to be more effective than NP-MSWL or GLP-1 monotherapy alone in achieving preoperative weight loss in this population. Patients in the mmAOM group achieved the highest mean %TBWL of 13.07%, compared to 8.14% in the Mono-GLP-1 group and 5.95% in the NP-MSWL group. Notably, the most substantial weight loss occurred ~51 weeks for the mmAOM group, suggesting that an extended period of pharmacological treatment in this high BMI group may be necessary to achieve maximal preoperative weight loss. Beyond 88 weeks, however, there may be diminishing returns. The observed plateau in weight loss beyond 88 weeks is consistent with prior research demonstrating that the effects of obesity treatments attenuate over time [11]. Several factors may contribute to this decline, including metabolic adaptation, medication adherence challenges, and patient-specific factors such as treatment fatigue or dose adjustments. Additionally, access to GLP-1 receptor agonists remains highly dependent on insurance coverage, and some patients may have discontinued therapy due to cost or formulary restrictions. Such considerations highlight the importance of long-term treatment planning and individualized approaches to obesity pharmacotherapy. Additionally, these data underscore the importance of a multimodal approach to obesity treatment, incorporating both pharmacological and operative strategies to achieve optimal outcomes in severe obesity cases. Specifically, the case highlighted in Fig. 3 is representative of the potential for substantial weight loss and BMI reduction using a staged neo-adjuvant therapeutic approach.

The overarching goal of preoperative weight loss is to optimize patients and reduce surgical complications, known benefits linked to improved postoperative outcomes. Even small amounts (e.g. average −3.3 kg) of preoperative weight loss significantly reduce hepatic volume and intra-abdominal adipose tissue, which reduces intraoperative difficulty during gastric bypass [12]. All groups in the present study achieved a median absolute pre-operative weight loss (NP-MSWL 7.8 kg, Mono-GLP-1 therapy 12.15 kg, and mmAOM 19 kg) that well-outpaced these prior findings, suggesting any intervention aimed at maximining preoperative weight loss could potentially improve the safety and efficacy of subsequent MS. Still et al. demonstrated in a retrospective study of 881 patients (mean BMI = 49 kg/m²) undergoing gastric bypass, that increasing amounts of preoperative weight loss were associated with reduced postoperative complications of all severity including major complications [13]. However, a systematic review of mostly retrospective, non-controlled studies, in patients with mean BMI 45–50 kg/m², was inconclusive as to whether preoperative weight loss was associated with lower postoperative complications [14]. While seemingly intuitive that preoperative weight loss for patients with high BMI (>70 kg/m²) would not only improve operability but also reduce postoperative complications, carefully controlled trials are necessary to answer this critically important question.

Notably, the superior efficacy of mmAOM therapy is consistent with emerging evidence of phenotypic contributions regarding individuals’ response to the treatment of obesity – a complex, multifaceted disease with various pathophysiological contributors [15]. Recent literature by Acosta and colleagues suggests that AOM responsiveness varies based on phenotypic characteristics (e.g. metabolic rate, behavioral factors) [16]. Thus, anti-obesity treatments may require tailoring to specific patient phenotypes to enhance treatment efficacy [16]. Moreover, studies have demonstrated that patients that align with multiple obesity phenotypes also have statistically higher body weight and BMI [17]. Thus, patients with a BMI ≥ 70 kg/m², who may be most likely to cluster into this poly-phenotypic group, may help explain why their response to mmAOM outpaced that of the most effective standalone AOMs – GLP-1RAs – a class of medications most often tested in populations with obesity that have a mean and median BMI < 40 kg/m^2^ [3, 18–24]. Future investigation into certain combinations obesity therapies could help in customizing treatment plans that leverage mmAOM for more significant weight loss outcomes.

This study is not without limitations. This single-institution setting and inherently finite sample size for analysis may limit generalizability of the findings, though higher BMI patients may be increasingly considered for metabolic surgery. Regardless, the incidence of higher BMI is not well-defined, as most scientific studies categorize severe obesity broadly as BMI > 40 or >50. Additionally, this study was retrospective in nature, and while efforts were made to control for confounders, unmeasured variables such as clinician prescribing preferences, medication adherence, and patient-specific factors may have influenced outcomes. Aside from this, access to medications and adherence to prescribed regimens can also be influenced by insurance coverage and supply chain stability, potentially further confounding our results. Thus, selection of specific pharmacologic agent was often based on what was available for the patient based on insurance coverage rather than what was most efficacious.

The observed differences in diabetes prevalence across treatment groups likely reflect real-world prescribing trends, where GLP-1 receptor agonists are preferentially used in patients with diabetes due to their dual role in glycemic and weight management. Additionally, insurance coverage constraints often dictate access to these medications, potentially introducing treatment bias within the study population. These factors should be considered when interpreting weight loss differences between groups.

In addition, magnitude of weight loss is well documented to be blunted in the presence of diabetes [25–27]. This study observed that diabetes was more common in the Mono-GLP-1 group, a class of medications that is more readily covered with concurrent diabetes, which may partially explain the diminished responses GLP-1-RA therapy. Furthermore, we did not assess long-term weight loss maintenance or post-surgical outcomes, which are important areas for future research.

Additionally, given the evolving efficacy profiles of newer GLP-1 receptor agonists, we performed a secondary analysis categorizing patients based on whether they had ever received a second-generation GLP-1-RA (semaglutide or tirzepatide). However, due to the retrospective nature of this study, many patients transitioned between agents at various points during treatment, often due to changes in insurance coverage, medication availability, or clinical response. As a result, patients were classified as second-generation GLP-1 users if they had been prescribed semaglutide or tirzepatide at any time, regardless of prior first-generation GLP-1-RA use. This approach ensures that the impact of these more potent agents is appropriately captured while acknowledging the inherent heterogeneity of real-world prescribing practices – but the results should be interpreted with caution.

To our knowledge, that compares mmAOM therapy to single GLP-1 agents and NP-MSWL. As such, it is the first to suggest the use of multiple AOMs, in appropriately identified patients, may provide superior pre-operative %TBWL. These results provide important insights into preoperative weight management strategies for high-BMI patients; however, future research should focus on prospective trials with standardized treatment protocols to further define the role of multi-modal AOM therapy. Controlled studies with defined medication regimens, fixed treatment durations, and long-term follow-up will be critical to determine the optimal sequencing and duration of pharmacologic therapy in the preoperative setting. As coverage expands for increasingly potent AOMs, developing evidence based, preoperative protocols and algorithms is crucial to maximize the effectiveness of obesity treatment. Future prospective studies with larger sample sizes and standardized protocols are needed to confirm these observations and address these confounding factors.

## Conclusion

Multi-modal anti-obesity medication treatment appears to be superior to non-pharmacologic medically supervised weight loss or GLP-1 monotherapy for preoperative weight loss in patients with BMI ≥ 70 kg/m^2^, suggesting that multiple AOMs in the appropriately selected patient may yield significantly greater pre-operative weight loss. This approach may be particularly beneficial for up to 12 months of treatment for intended preoperative weight loss in high BMI patients, after which %TBWL may begin to wane. Future research should focus on larger, multi-center trials to validate these findings, explore the long-term efficacy of this approach, and its impact on surgical outcomes. Additionally, further translational investigation into phenotyping in clinical practice may help identify patients most suitable for specific obesity therapies, enhancing the precision of obesity management.

## Supplementary information

Supplemental Table 1 - Weight Loss Outcomes by Treatment Group & Diabetic Status

Supplemental Table 2 - Weight Loss Outcomes by Treatment Group & GLP-1 Generation

## Data Availability

The data that support the findings of this study are not publicly available due to the risk of participant re-identification, even after de-identification measures, given the detailed nature of the dataset. Data are available from the corresponding author upon reasonable request and for non-commercial research purposes, contingent on approval by the appropriate institutional review board and in compliance with confidentiality agreements. Aggregated and composite data used for reporting are available within the manuscript.
