# Supplementary material for: Multi-modal neo-adjuvant anti-obesity medications may be more effective than medically supervised weight loss or GLP-1 therapy alone in preparing BMI≥70 patients for metabolic surgery
Source: Int J Obes (Lond). 2025 Jun 2;49(8):1516–22. doi: 10.1038/s41366-025-01798-2 (PMC12396963; doi:10.1038/s41366-025-01798-2)
Supplement: Supplementary file 1 — Supplemental Table 1 - Weight Loss Outcomes by Treatment Group & Diabetic Status [file 41366_2025_1798_MOESM1_ESM.docx]

Supplemental Table 1 - Weight Loss Outcomes by Treatment Group & Diabetic Status

|  |  | Diabetes | No Diabetes | p-value |
| --- | --- | --- | --- | --- |
| Overall | Total, n | 69 | 44 |  |
|  | %TBWL, median [IQR] | 7.19 [4.11-11.64] | 8.63 [3.45-15.47] | 0.467 |
|  | Δ BMI, median [IQR] | 5.96 [3.87-10.52] | 7.92 [2.95-12.55] | 0.689 |
|  | Δ kg, median [IQR] | 14.3 [6-24.3] | 16.55 [5.07-34.62] | 0.739 |
|  | %EWL, median [IQR] | 10.83 [5.88-17.02] | 12.95 [4.97-21.46] | 0.475 |
| NP-MSWL | Total, n | 4 | 9 |  |
|  | %TBWL, median [IQR] | 3.37 [2.58-4.31] | 6.12 [4.23-7.87] | 0.214 |
|  | Δ BMI, median [IQR] | 4.82 [3.86-5.32] | 6.15 [4.16-12.93] | 0.503 |
|  | Δ kg, median [IQR] | 6.8 [5.48-8.65] | 8.3 [5-16.1] | 0.503 |
|  | %EWL, median [IQR] | 4.82 [3.64-6.16] | 8.77 [6.38-11.48] | 0.154 |
| Mono-GLP-1 | Total, n | 37 | 17 |  |
|  | %TBWL, median [IQR] | 5.61 [2.64-12.1] | 5.56 [1.76-10.96] | 0.558 |
|  | Δ BMI, median [IQR] | 5.51 [3.12-10.52] | 4.05 [1.49-9.88] | 0.31 |
|  | Δ kg, median [IQR] | 12.6 [4.9-28.4] | 10.8 [4.2-28.8] | 0.852 |
|  | %EWL, median [IQR] | 8.23 [3.88-17.66] | 8.42 [2.5-15.92] | 0.585 |
| mmAOM | Total, n | 28 | 18 |  |
|  | %TBWL, median [IQR] | 9.56 [6.68-11.36] | 14.03 [8.23-21.06] | 0.072 |
|  | Δ BMI, median [IQR] | 7.1 [5.15-11.21] | 10.61 [6.63-14.47] | 0.222 |
|  | Δ kg, median [IQR] | 18.01 [13.72-24.8] | 26.5 [11.18-42.05] | 0.322 |
|  | %EWL, median [IQR] | 13.14 [9.66-16.69] | 20.17 [12.11-31.88] | 0.076 |

Abbreviations: NP-MSWL = Non-Pharmacologic Medically Supervised Weight Loss; Mono-GLP-1 = Glucagon-Like Peptide-1 Receptor Agonist Monotherapy (Mono-GLP-1); mmAOM = Multi-Modal Anti-Obesity Medication, BMI = Body Mass Index. All data are presented as mean [95% confidence interval]. Percent Total Body Weight Loss (%TBWL) calculated as: %TBWL = ((Presentation Weight − Post-treatment Weight)) / Initial Weight) × 100. Percent Excess Weight Loss (%EWL) calculated as: %EWL = (Weight Loss) / (Presentation Weight − Ideal Weight) × 100. Ideal weight was based on a BMI of 25 kg/m².
