# Supplementary material for: Multi-modal neo-adjuvant anti-obesity medications may be more effective than medically supervised weight loss or GLP-1 therapy alone in preparing BMI≥70 patients for metabolic surgery
Source: Int J Obes (Lond). 2025 Jun 2;49(8):1516–22. doi: 10.1038/s41366-025-01798-2 (PMC12396963; doi:10.1038/s41366-025-01798-2)
Supplement: Supplementary file 2 — Supplemental Table 2 - Weight Loss Outcomes by Treatment Group & GLP-1 Generation [file 41366_2025_1798_MOESM2_ESM.docx]

Supplemental Table 2 - Weight Loss Outcomes by Treatment Group & GLP-1 Generation

|  |  | Second_Gen_GLP1 | First_Gen_GLP1 | NO_GLP | p-value |
| --- | --- | --- | --- | --- | --- |
| Overall | Total, n | 79 | 8 | 26 |  |
|  | %TBWL, median [IQR] | 9.05 [4.77-13.46] | 4.44 [3.18-5.87] | 5.73 [3.07-9.39] | 0.048 |
|  | Δ BMI, median [IQR] | 7.73 [3.89-11.83] | 3.71 [2.57-6.28] | 5.82 [3.17-8.72] | 0.338 |
|  | Δ kg, median [IQR] | 17.5 [9.32-32.95] | 9.7 [6.33-12.95] | 9.8 [5.02-18.23] | 0.049 |
|  | %EWL, median [IQR] | 13.09 [7.14-20.45] | 6.44 [4.63-8.14] | 8.75 [4.53-13.49] | 0.043 |
| NP-MSWL | Total, n | – | – | 13 |  |
|  | %TBWL, median [IQR] | – | – | 5 [2.74-6.79] | – |
|  | Δ BMI, median [IQR] | – | – | 5 [4.16-6.28] | – |
|  | Δ kg, median [IQR] | – | – | 7.8 [5-15.5] | – |
|  | %EWL, median [IQR] | – | – | 7.2 [4.02-9.35] | – |
| Mono-GLP-1 | Total, n | 50 | 4 | – |  |
|  | %TBWL, median [IQR] | 6.16 [2.52-12] | 3.45 [3.1-4.01] | – | 0.257 |
|  | Δ BMI, median [IQR] | 5.35 [1.95-10.66] | 2.83 [2.48-4.02] | – | 0.241 |
|  | Δ kg, median [IQR] | 12.7 [4.23-29.18] | 6.8 [6.33-8.25] | – | 0.372 |
|  | %EWL, median [IQR] | 9.03 [3.75-17.54] | 4.97 [4.51-5.7] | – | 0.192 |
| mmAOM | Total, n | 29 | 4 | 13 |  |
|  | %TBWL, median [IQR] | 10.81 [8.48-16.66] | 5.91 [4.67-6.72] | 8.84 [3.94-11.28] | 0.033 |
|  | Δ BMI, median [IQR] | 9.82 [6-13.18] | 5.21 [3.88-10.41] | 7.89 [3.05-8.95] | 0.195 |
|  | Δ kg, median [IQR] | 23 [16.4-37.9] | 13.3 [10.6-15.25] | 14.3 [5.1-26.7] | 0.061 |
|  | %EWL, median [IQR] | 15.64 [12.93-23.87] | 8.36 [6.84-9.45] | 13.21 [5.97-16.84] | 0.039 |

Abbreviations: NP-MSWL = Non-Pharmacologic Medically Supervised Weight Loss; Mono-GLP-1 = Glucagon-Like Peptide-1 Receptor Agonist Monotherapy (Mono-GLP-1); mmAOM = Multi-Modal Anti-Obesity Medication, BMI = Body Mass Index. All data are presented as mean [95% confidence interval]. Percent Total Body Weight Loss (%TBWL) calculated as: %TBWL = ((Presentation Weight − Post-treatment Weight)) / Initial Weight) × 100. Percent Excess Weight Loss (%EWL) calculated as: %EWL = (Weight Loss) / (Presentation Weight − Ideal Weight) × 100. Ideal weight was based on a BMI of 25 kg/m².
